# *Bacillus subtilis* Feed Supplementation Combined with Oral *E. coli* Immunization in Sows as a Tool to Reduce Neonatal Diarrhea in Piglets

**DOI:** 10.3390/ani14131978

**Published:** 2024-07-04

**Authors:** Jianxin Liu, Danchen Aaron Yang, Haobo Qu, Dandan Liu, Kehe Huang

**Affiliations:** 1 College of Veterinary Medicine, Nanjing Agricultural University, Nanjing 210095, China; 2 Institute of Animal Nutritional Health, Nanjing Agricultural University, Nanjing 210095, China

**Keywords:** piglet diarrhea, *Bacillus subtilis*, *Escherichia coli* feedback feeding, maternal antibody

## Abstract

**Simple Summary:**

*Escherichia coli* is one of the most common pathogens that causes piglet diarrhea, which poses a significant threat to the pig industry. Immunizing piglets through feedback feeding (i.e., feeding a homogenate of intestinal contents and tissues from *E. coli*-infected piglets to sows) has become a common strategy for preventing *E. coli*-induced diarrhea in suckling piglets. The uneven maternal antibody levels in sows after feedback feeding may be the main reason for the erratic effectiveness of this treatment. *Bacillus subtilis* (*B. subtilis*) is a kind of probiotic with excellent properties that can increase the levels of IgG and IgA in serum or milk. In this study, we aimed to investigate the effects of *B. subtilis* on the specific immune response of sows to *E. coli* and the diarrhea rate in their offspring. The data indicated that feeding *B. subtilis* to sows subjected to feedback feeding during late pregnancy and lactation could enhance their specific maternal immune response, which, in turn, could lead to better passive immunity in their offspring, resulting in an increased number of surviving weaned piglets, litter weight gain from birth to weaning, immunity, intestinal barrier function, and intestinal flora composition, as well as a decreased rate of diarrhea induced by *E. coli*.

**Abstract:**

To investigate the effects of *B. subtilis* on the specific immune response of lactating sows to *E. coli* and the diarrhea rate in suckling piglets, thirty large white sows with similar farrowing dates were randomly divided into two groups: a feedback feeding (i.e., feeding a homogenate of intestinal contents and tissues from *E. coli*-infected piglets to sows; FB) group and a feedback feeding with *B. subtilis* (FB + BS) group. Serum, colostrum, and intestinal tissues from sows and piglets were collected to assess the immune response and intestinal barrier function at weaning. T and B cells from Peyer’s patches (PPs) and mesenteric lymph nodes (MLNs) in lactating mice (with treatments consistent with the sows’) were isolated to explore the underlying mechanism. The results showed that, compared with the FB group, the reproductive performance of sows and the growth performance of their offspring were effectively improved in the FB + BS group. Moreover, the levels of IgG/IgA and those of IgG/IgA against *E. coli* in the serum and colostrum of sows in the FB+BS group were increased (*p* < 0.05). Meanwhile, the ratio of CD4+/CD8+, CD4+CXCR5+PD1+, and B220+IgA+ cells in MLNs and PPs, and the IgA levels in the mammary glands of mice, were also increased in the FB + BS group (*p* < 0.05). Notably, in suckling piglets in the FB + BS group, the diarrhea rate was decreased (*p* < 0.05), and the intestinal barrier function and intestinal flora composition at weaning were significantly improved. Overall, these results indicated that *B. subtilis* feed supplementation combined with feedback feeding in pregnant and lactating sows can reduce diarrhea in suckling piglets by enhancing the maternal immune response against *E. coli* and intestinal barrier function in their offspring, improving survival rates and pre-weaning growth.

## 1. Introduction

Currently, neonatal porcine diarrhea caused by bacteria is the main problem in some large-scale breeding farms free of enteroviruses [1], with diarrhea induced by *E. coli* being the most common form [2,3,4]. This disease causes high mortality or low weight gain in suckling piglets, leading to severe economic losses for the pig industry [5,6,7]. Reducing the pathogen load of bacteria in the environment and the sow body surface through disinfection, washing, and enhancing the immunity of piglets against pathogens through vaccination are the main prevention and control measures [8,9]. However, it is difficult to synthesize a strain-specific vaccine with efficient cross-protection because of the serotype diversity of *E. coli* strains [1,10]. Therefore, sows are often fed a homogenate of intestinal tissues and contents from piglets who succumbed to diarrhea to stimulate their immune system and pass specific antibodies to suckling piglets through colostrum and milk when *E. coli*-induced diarrhea occurs in pig farms, which is also known as feedback feeding [9,11,12]. However, the individual differences in the immune response of the same batch of sows to feedback feeding make it impossible to achieve consistent results from this procedure.

The gastrointestinal tract is one of the largest immunological organs of the body [13]. Feedback feeding is a kind of gastrointestinal mucosal immune strategy. Mucosal immunity provides the first line of defense against exogenous pathogens by secreting SIgA and IgM to inhibit surface colonization by microorganisms [13,14,15]. B cells in mucosa-associated lymphoid tissue are the cells that give rise to secretory IgA and IgM. During the perinatal period, in sows, maternal IgA-secreting cells migrate to the mammary gland to increase the levels of maternal antibodies in milk [15]. The authors of a previous study verified the involvement of intestinal microorganisms in the number of IgA plasma cells in the mammary glands (but not the small and large intestines), as well as the production of maternal IgA in milk [16].

*B. subtilis* is a kind of probiotic with excellent properties that can be widely used as a feed additive in livestock and poultry farming [17,18,19]. It has been shown that *B. subtilis* can increase the levels of IgG, IgM, and SIgA in serum or milk [20,21]. In this study, we aimed to enhance the immune response of sows against *E. coli* by combining *B. subtilis* with feedback feeding to increase the levels of specific maternal antibodies to improve intestinal function and reduce *E. coli*-caused diarrhea in suckling piglets. Our study provides a new resolution for the prevention and control of *E. coli*-caused diarrhea in suckling piglets.

## 2. Materials and Methods

### 2.1. Ethical Approval

All experiments on animals were carried out according to the Ethical Guidelines of Laboratory Animals in Research, National Research Council, and were approved by the Animal Care and Use Committee, Nanjing Agricultural University, according to the Guidelines for the Administration of Laboratory Animals (Jiangsu Province). The ethical code of the pig experiment is NJAULLSC2022015, and that of the mouse experiment is NJAU.No20230309019.

### 2.2. Feedback Feeding

Feedback feeding materials were obtained from intestinal samples from *E. coli*-infected 3-day-old piglets. First, diarrhea piglets without antibiotic treatment were euthanized to obtain intestinal tissues and contents; then, the homogenate of intestinal tissues and contents were added to the feed of sows 14 and 21 days before delivery, under the guidance of a veterinarian [12,22]. Specifically, the intestine from a euthanized piglet was homogenized, and 4 L of distilled water and 200 g of milk replacer were added. This amount of intestinal homogenate could be fed to approximately 20 sows, which were orally administered 200 mL of homogenate each. Moreover, the risk of infection by pathogens other than *E. coli* was eliminated by employing polymerase chain reaction (PCR) detection.

### 2.3. Animal Management and Experiment Design

The pig experiment was performed on a GGP breeding pig farm with 1250 sows in Liaoning Province, China, from November 2022 to January 2023. Thirty large pregnant Yorkshire sows were enrolled in this trial and housed in individual crates; they were second or third parity sows and had a similar body condition, neither too thin nor too fat, with a body weight of 220–260 kg. Thirty sows were randomly divided into two groups with 15 sows in each group. Sows in the control group (FB group) were treated with the homogenate of intestinal tissues and contents three weeks before delivery. Sows in the experimental group (FB + BS group) were administered not only the homogenate, similarly to the control group, but also a 5 g *B. subtilis* spore (2 × 10^8^ CFU/g) supplement per day with their feed from two weeks before homogenate treatment to weaning day (the 21st day after birth).

These sows were all fed 1.8 kg/day gestation feed (Cargill; Table 1) in the morning and had access to water through nipple drinkers ad libitum. They were transferred to farrowing pens in the farrowing room 2 days before the expected farrowing date. Thereafter, they were fed 2 kg/day lactating feed until farrowing. During the lactation period, feed was provided daily in the range of 1 kg to maximum consumption during the first week and until weaning. Sows were fed with an automatic feeder. In the farrowing room, sows were cared for by day- and night-shift staff, so that a delivery assistant was immediately available when necessary.

At farrowing, technicians documented and calculated the litter size, number of piglets born alive, number of stillborn piglets, number of mummies, litter weight at birth, and live-born piglet weight, and provided assistance when necessary. The piglets that were not breathing and had brown or dark skin were defined as mummies, and others were defined as stillborn. The litter size was standardized to 13 piglets per litter within the two groups to balance the numbers of sow teats and piglets after the latter started suckling colostrum (6–24 h after birth). Other operations, such as ear tagging, tail cutting, iron injection, castration, coccidiosis prevention, and vaccination, were performed as part of the common management of the farm. Before weaning, piglets had free access to a water supply through nipple drinkers while without a feed supply. On the weaning day (the 21st day after birth), technicians documented and calculated the number of weaned piglets per litter, litter weight at weaning, litter weight gain from birth to weaning, piglet weight at weaning, piglet daily weight gain from birth to weaning, and piglet survival rate.

The mouse experiment was performed in the laboratory animal center of Nanjing Agricultural University. Twenty pregnant C57BL/6 mice (12 weeks old) were purchased from Gempharmatech Co., Ltd., Nanjing China, and were randomly divided into the control group (FB group) and the experimental group (FB + BS group), with ten mice per group. FB + BS mice were given 200 μL of *B. subtilis* suspension solution every day for 14 days by gavage, and FB mice were given 200 μL of saline solution. Then, all of the mice were challenged with *E. coli* (1 × 10^8^) for three consecutive days on day 7 before delivery.

### 2.4. Diarrhea Rate Assessment

The litter diarrhea rate was calculated based on the number of piglets with diarrhea in each litter and was used for calculating the group diarrhea rate. The litter diarrhea rate was defined as the ratio of the accumulated number of diarrhea piglets within 7 days after birth to the initial litter size after cross-fostering. Piglets with yellow, loose stool around the anus were identified as having diarrhea and marked with a marker. The same piglet would not be counted repeatedly, even if these symptoms were found on different days.

### 2.5. Sample Collection

Blood samples for serum separation were collected in anticoagulant-free vacutainers from the jugular vein of sows at the following time points: on the day before feedback feeding; and 7, 14, and 21 days after feedback feeding. The blood samples were collected in 4 steps, as per the following procedure: (1) sows were restrained by the hog holder; (2) the jugular fossa was disinfected with 75% alcohol; (3) a blood collection needle (#18G 1/2″) was inserted vertically into the skin; (4) the needle was connected to the blood collection tube. The collected blood samples were centrifuged at 1000× *g* for 15 min at 4 °C, and the separated sera were stored at −80 °C. Colostrum samples (10 mL per sow) were collected from the sows when they exhibited signs of delivery. Before collecting, the udders were cleaned with warm water and disinfected with 75% alcohol. The first 3–5 drops were discarded to avoid outside microbial contamination. All of the samples were stored at −20 °C until further component analysis.

Five piglets were randomly selected from each group for sample collection on the day of weaning. Blood samples, intestinal contents, and small intestinal tissues were collected from the piglets after they were euthanized with phenobarbital sodium injection, which was performed according to the following 2 steps: (1) The piglets were subjected to intramuscular injection with 5% phenobarbital sodium at a dose of 200 mg/kg. (2) When the piglets showed deep anesthesia, they were placed in the supine position, and their blood was collected through the anterior vena cava by using an 18 G 1/2″ needle; then, the piglets were placed in the side-lying position, and one of the front legs was lifted to expose the armpit, which allowed for the cutting off of their axillary arteries and veins and the subsequent bleeding out of the animals. The blood samples were centrifuged to separate the serum. Then, all of the samples were stored at −80 °C, and a portion of the small intestinal tissues was fixed in 4% paraformaldehyde for pathological section processing.

All lactating mice were euthanized within three days of delivery for the collection of blood samples, Peyer’s patches (PPs), mesenteric lymph nodes (MLNs), and mammary gland tissues. The serum separation procedures for mice were the same as those mentioned earlier. All samples were stored at −80 °C, and a portion of the mammary gland tissue was fixed in 4% paraformaldehyde for pathological section processing.

### 2.6. Antibody Level Analysis

The levels of IgA and IgG against *E. coli* in sow serum and colostrum were measured using porcine ELISA kits (Abcam, USA), following the manufacturer’s instructions. Similarly, the levels of total IgA and total IgG in the serum or colostrum of sows, mice, or piglets were measured using porcine ELISA kits (MyBioSource, USA) following the manufacturer’s instructions. 

### 2.7. Intestinal Tissue Histopathological Determination

The intestinal tissues were fixed in paraformaldehyde, dehydrated in a series of alcohol concentrations (70%, 80%, 90%, and 100%), then embedded in paraffin. The tissue sections were stained with hematoxylin-eosin (HE) and measured under a microscope at a combined magnification of 40× using an image processing and analysis system (Leica Imaging Systems Ltd., Cambridge, UK). The villus height (VH):crypt depth (CD) ratio (VH: CD) was measured using the Image-pro Plus 6.0 program (Media Cybernetics, Inc., Georgia, USA). A total of 25 villous samples with intact lamina propria were randomly selected and blindly examined for measurement.

### 2.8. Intestinal Tissue Tight Junction Proteins and Mucin Level Detection

The mRNA levels of *ZO-1*, *occludin*, *claudin 1*, and *muc-2* in intestinal tissue were measured using RT-qPCR. Total RNA was extracted from the piglet intestinal tissues using RNA extraction-assisted reagent (Accurate Biology, Changsha, China) and converted into cDNA using the Evo M-MLV Reverse Transcription Kit (Accurate Biology, Changsha, China), following the manufacturer’s protocol. Taqman primers for qRT-PCR were synthesized by Sangon Biotech (Shanghai, China) and are listed in Table 2. β-actin was used for normalization. The difference in mRNA expression was determined according to delta CT against β-actin. All real-time quantitative PCR studies were run in triplicate.

The levels of *ZO-1* in the intestinal tissues were measured with immunofluorescence. The intestinal sections were subjected to deparaffinization in xylene, rehydration with a graded alcohol series, and washing with a 0.1 M citric acid solution. Next, the slides were incubated with 10% bovine serum albumin for 30 min to block the non-specific antigens and then stained with the primary antibody (Cell Signaling Technology, Inc., Danvers, MA, USA) against *ZO-1* (1:400) for 6 h at 4 °C, followed by FITC-conjugated goat anti-mouse IgG (1:2000) and DAPI (1 μL/mg) for 1 h at 37 °C. Images and fluorescence signals were captured with a fluorescence microscope (Olympus, Tokyo, Japan).

### 2.9. Mammary Tissue Histopathological Changes and IgA Protein Level Detection

The histopathological changes in mammary tissue were measured with hematoxylin-eosin (H&E) staining. After the mice were euthanized, their mammary tissues were collected and fixed in 10% formalin. Following xylene dewaxing and gradient ethanol hydration, the tissues were embedded in paraffin and cut into 4 µm-thick sections, which were then stained with H&E and observed with a binocular Olympus CX31 microscope.

The levels of IgA in the mammary tissues were measured by using immunohistochemistry. The procedures before the incubation with the primary antibody were consistent with the immunofluorescence process described above. The slides were then stained with the primary antibody (Cell Signaling Technology, Inc., Danvers, MA, USA) against IgA (1:5000) for 2 h at 37 °C, followed by the secondary antibody for 1 h at 37 °C. The relative expression levels of the target proteins were analyzed with the Image-Pro Plus 6.0 program (Media Cybernetics, Rockville, MD, USA).

### 2.10. Intestinal Microbiota Analysis

The total DNA in each stool sample from both groups was extracted with the Stool Total DNA Extraction Kit (TIANGEN, Beijing, China), following the manufacturer’s instructions. The integrity of the extracted genomic DNA was assessed with electrophoresis on a 1% (*w*/*v*) agarose gel. An Illumina HiSeq PE250 (Illumina, San Diego, CA, USA) was used to perform 16S rDNA high-throughput sequencing. The amplification of the V3–V4 variable region of 16S rDNA was performed with primers (341F-806R). The raw data were subjected to quality control by using UPARSE. The operational taxonomic units (OTUs) at 97% similarity were generated by clustering qualified reads [22]. Three indices of alpha diversity (Chao1, Shannon, and Simpson) in QIIME software (v2.0) were used to analyze the species diversity of each sample. Principal component analysis (PCA), principle coordinates analysis (PCoA), Venn diagram, and species abundance analysis were performed by using R version 3.3.0. The linear discriminant analysis effect size method (http://huttenhower.sph.harvard.edu/lefse/; accessed on 10 March 2024) was used to analyze the microbial features.

### 2.11. Flow Cytometry Analysis

Immediately after sacrifice, Peyer’s Patches (PPs) and mesenteric lymph nodes (MLNs) were collected, ground, and filtered through 100 μm cell strainers (BD Biosciences). Cells from PPs were divided into two parts and then stained with antibodies as follows: (a) anti-CD3-FITC (11-0031-81, eBioscience, Waltham, MA, USA), anti-CD4-PE (25-0042-81, eBioscience), anti-CD8-APC (17-0081-82, eBioscience), and anti-CD19-PE (12-0193-81, eBioscience); (b) anti-CD3FITC (11-0031-81, eBioscience), anti-CD4-PE (25-0042-81, eBioscience), anti-CD185-APC (17-7185-80, eBioscience), and anti-CD279-PE (12-9985-81, eBioscience). Cells from MLNs were divided into two parts and then stained with antibodies as follows: (a) anti-CD3-FITC (11-0031-81, eBioscience), anti-CD4-PE (25-0042-81, eBioscience), anti-CD185-APC (17-7185-80, eBioscience), and anti-CD279-PE (12-9985-81, eBioscience); (b) anti-CD19-PE (12-0193-81, eBioscience), anti-CD45R-APC (17-0452-82, eBioscience), and anti-IGA-FITC (11-4204-81, eBioscience). The cell surface staining lasted for 30 min at 4 °C in the dark. Then, the cells were centrifuged and resuspended with 100 μL of PBS for analysis. Single-cell suspensions were examined using a flow cytometer (BD Biosciences, San Jose, CA, USA), and the data were analyzed using FlowJo software (v10.0).

### 2.12. Statistical Analysis

All data were analyzed using R language, version 4.3.0 (R Core Team, Auckland, NZ, 2014), except for those analyzed using the chi-squared test and Wilcoxon rank-sum test (where stated). The treatment effect on the continuous phenotypic data (e.g., litter weight at birth) was analyzed using a *t*-test (absence of heteroscedasticity suggested by an F test). The normality assumption was visually examined by using a q-q plot based on the studentized residuals. For discrete phenotypic data (e.g., number of stillborn piglets/total births), logistic regression models were fitted to compare the effect between the two groups. If the denominators were equal across the litters (i.e., survived proportion and diarrhea proportion), a chi-squared test was performed (SPSS version 26). For the antibody data, the Wilcoxon rank-sum test (SPSS version 26) was applied to compare the antibody levels at a given time point between the two groups. When the antibodies were measured over time, they were first log-transformed, and multiple linear models were created, with treatment, time, and product term (interaction) being included as predictors. The presence of an interaction was decided based on the likelihood ratio test. The multiple linear models were deemed parsimonious, as linear mixed models were also fitted for comparison purposes (the clustering effect was minimal). The homogeneity and normality assumptions were visually examined by studentized residuals against fitted values plots and q-q plots, respectively.

## 3. Results

### 3.1. B. subtilis Improved the Reproductive Performance of Sows Challenged with E. coli and the Growth Performance of their Offspring

On the day of delivery, the effects of *B. subtilis* on the reproductive performance of sows challenged with *E. coli* were assessed. Compared to the FB group, the number of piglets born alive increased when *B. subtilis* was added to the feed (FB + BS group) (odds ratio [OR] = 2.77, 95% confidence interval [CI]: 1.45–5.6). Therefore, FB + BS decreased the odds of having stillborn (OR 0.43, 95%CI 0.17–0.98) and mummified piglets (OR 0.32, 95%CI 0.1–0.85). In addition, the litter weight at birth was observed to be higher in the FB + BS group. However, BS had no significant effects on the litter size (total births) or the live-born piglet weight (*p* > 0.05) (Table 3).

On the day of weaning, the growth performance of the piglets was assessed. As shown in Table 3, compared with the FB group, the number of weaned piglets, litter weight at weaning, and litter weight gain from birth to weaning in the FB + BS group were significantly increased (*p* < 0.05). There were no significant differences in individual piglet body weight at weaning or daily piglet weight gain during lactation (*p* > 0.05). Importantly, *B. subtilis* significantly reduced the rate of diarrhea in suckling piglets (*p* < 0.05).

### 3.2. B. subtilis Enhanced the Immune Response of Sows to E. coli

**Figure 1:**
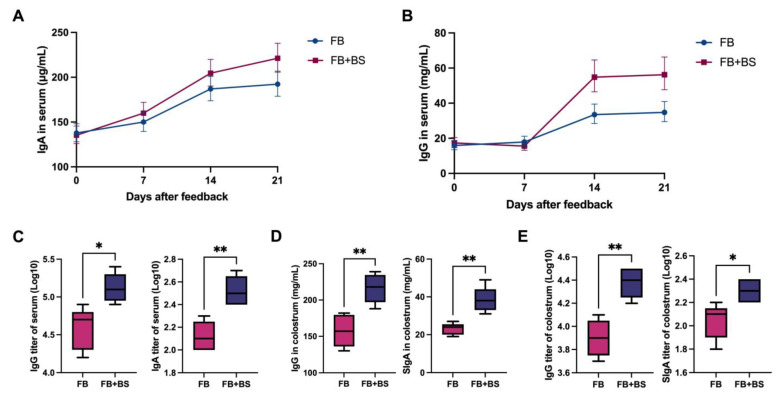
Effects of *B. subtilis* on the serum and colostrum antibody levels of sows after the *E. coli* challenge. (**A**) The concentrations of IgA in serum (geometric mean) were measured by ELISA; (**B**) The concentrations of IgG (geometric mean) in serum were measured by ELISA; (**C**) The concentrations of F4ac-IgG and F4ac-IgA in serum were measured by ELISA; (**D**) The concentrations of IgG and IgA in colostrum were measured by ELISA; (**E**) The concentrations of F4ac-IgG and F4ac-IgA in colostrum were measured by ELISA. Data are shown as medians (IQR), N = 5. * *p* < 0.05 and ** *p* < 0.01 were considered statistically significant.

To investigate the mechanism of *B. subtilis* in reducing diarrhea in suckling piglets caused by *E. coli*, the levels of IgA/IgG and those of specific IgA/IgG against *E. coli* in the sow serum and colostrum were measured. As shown in Figure 1A, there were no differences in the IgA concentrations in sow serum between the two groups before and 14 days after the *E. coli* challenge (0 d/7 d and 14 d). Fourteen days after the *E. coli* challenge, there was a significant increase in IgG concentrations in the serum of sows in the FB + BS group (Figure 1B). Additionally, the antibody concentrations in the colostrum of the FB + BS group were significantly increased (*p* < 0.05) compared with the FB group (Figure 1D). More importantly, the levels of the specific IgA and IgG antibodies against *E. coli* in the serum (Figure 1C) and colostrum (Figure 1E) of sows in the FB + BS group were also significantly increased (*p* < 0.05).

### 3.3. IgA-Producing Plasma Cell Accumulation in the Intestinal Lymph Nodes and Transfer to Mammary Tissues might Participate in the Immune Response Enhanced by B. subtilis

**Figure 2:**
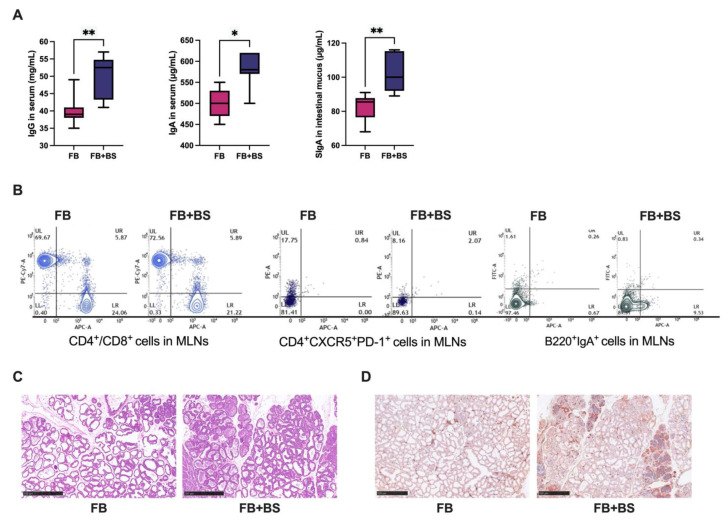
Effects of *B. subtilis* on the antibody levels and IgA-producing plasma cell accumulation of mice after the *E. coli* challenge. (**A**) Concentrations of IgG and IgA in serum and SIgA in intestinal mucus were measured by ELISA; (**B**) MLN and PP representative flow cytometry plots (including percentages) of CD4+CD8+ cells, CD4+CXCR5+PD1+ cells, and B220+IgA+ cells; (**C**) H&E staining of the mammary tissue (scale bar = 500 μm); (**D**) Immunohistochemical diagram of IgA in mammary tissue (scale bar = 500 μm). Data are shown as medians (IQR), N = 5. * *p* < 0.05 and ** *p* < 0.01 were considered statistically significant.

Pregnant mice experiments were performed to further elucidate the mechanism. Consistent with the sow experiments, the serum concentrations of IgA and IgG in the serum and SIgA in intestinal mucus of lactating mice in the FB + BS group were significantly increased (Figure 2A, *p* < 0.05). T cells and B cells from Peyer’s patches (PPs) and mesenteric lymph nodes (MLNs) in lactating mice were isolated to explore the mechanism of the increased maternal antibody levels. The results of the flow cytometry analysis showed that, compared with the FB group, the ratio of CD4+/CD8+ and numbers of CD4+CXCR5+PD1+ cells, and B220+IgA+ cells in the MLNs and PPs, were significantly increased in the FB + BS group (Figure 2B). Moreover, there were no significant differences in the pathological changes in the mammary gland tissues between the two groups (Figure 2C). However, the expression of IgA in the mammary glands of mice in the FB + BS group was significantly higher than that in the FB group (Figure 2D, *p* < 0.05).

### 3.4. B. subtilis Enhanced the Immune Response and Intestinal Barrier Function of Weaned Piglets Born to E. coli-Challenged Sows

**Figure 3:**
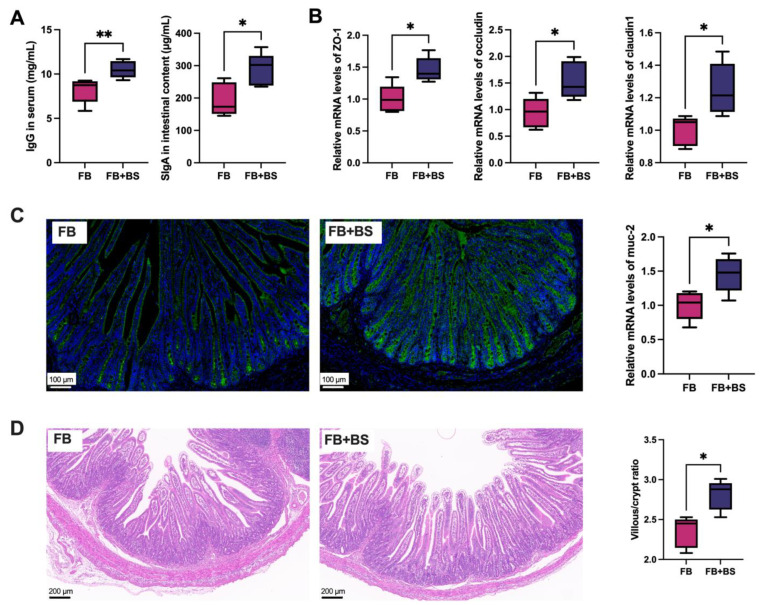
Effects of *B. subtilis* on serum antibody levels and intestinal barrier function of piglets born to *E. coli*-challenged sows. (**A**) The concentrations of IgG and IgA in serum were measured by ELISA; (**B**) The mRNA levels of *ZO*-*1*, *occludin*, *claudin1*, and *muc*-*2* were measured by RT-qPCR; (**C**) The immunofluorescence diagram of *ZO*-*1* in intestinal tissue, (scale bar = 200 μm); (**D**) The H&E staining of the intestinal tissue, (scale bar = 200 μm). The data are shown as median (IQR), N = 5. * *p* < 0.05 and ** *p* < 0.01 were considered statistically significant.

The intestinal function of weaned piglets was assessed. As shown in Figure 3A, compared with the FB group, the concentrations of IgG in serum and SIgA in intestinal content of piglets in the FB + BS group increased significantly (*p* < 0.05). Moreover, the intestinal barrier function of piglets from the FB + BS group was improved significantly by increasing the mRNA (Figure 3B) levels of tight junction proteins (*ZO*-*1 occludin* and *claudin1*) and *muc*-*2* (*p* < 0.05), as well as the *ZO*-*1* protein expression (Figure 3C). Meanwhile, the ratio of intestinal villi length to crypt depth in piglets from the FB + BS group also increased significantly (Figure 3D, *p* < 0.05).

### 3.5. B. subtilis Increased the Gut Microbiota Diversity of Suckling Piglets Born to E. coli-Challenged Sows

**Figure 4:**
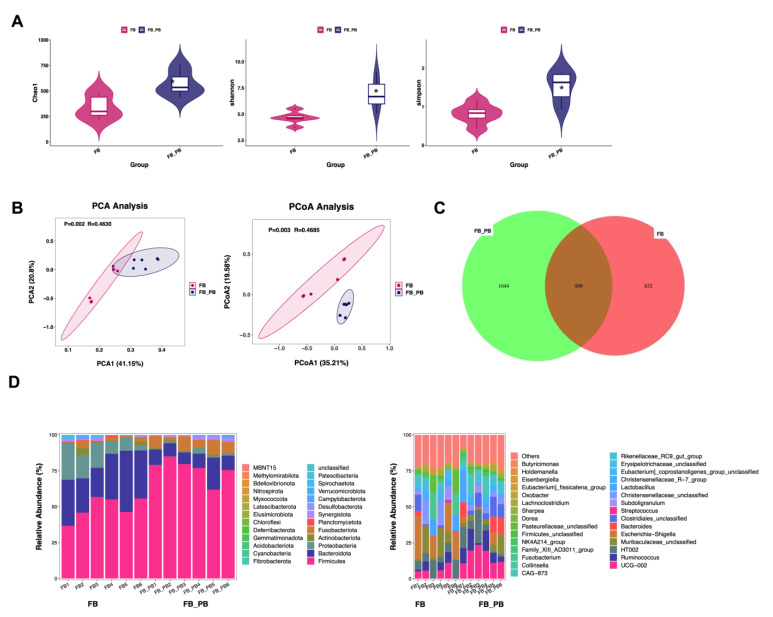
Effects of *B. subtilis* on the gut microbiota diversity of suckling piglets born to *E. coli*-challenged sows. (**A**) Fecal microbial alpha diversity at the species level was estimated by the Chao1 index, Shannon index, and Simpson index; (**B**) PCA and PCoA analysis of piglet rectum content microbiome; (**C**) Venn diagram displaying group overlaps; (**D**) Average relative abundance of the dominant phylum and genus in each group. Data are shown as median (IQR), N = 5. * *p* < 0.05 was considered statistically significant.

Fresh cecum contents were collected and analyzed with 16S rDNA sequencing. The results of the intestinal flora α diversity are shown in Figure 4A. The indexes of Chao1 value, Shannon value, and Simpson value in the FB + BS group were significantly higher than those in the FB group (*p* < 0.05), indicating that flora richness and evenness in the FB + BS group were higher than those in the FB group. The PCA and PCoA analyses indicated that the bacterial community compositions in the FB + BS and FB groups were significantly different (Figure 4B). Moreover, the Venn diagram displaying group overlaps indicated that 1044 species of the total richness of 1553 species were unique to the FB + BS group (Figure 4C). The results of the intestinal flora community structure are shown in Figure 4D. *Bacteroidota*, *Firmicutes*, and *Proteobacteria* were the dominant bacteria in both the FB and FB + BS groups, accounting for 96.24% and 85.84% of the total bacteria, respectively. At the phylum level, *Firmicutes* and *Bacteroides* were the dominant phyla in both groups, with the relative abundance of *Firmicutes*, *Bacteroidota*, and *Synergistota* being higher and the relative abundance of *Proteobacteria*, *Actinobacteriota*, *Fusobacteriota*, *Planctomycetota*, and *Spirochaetota* being lower in the FB + BS group (Figure 4D). At the genus level, the top three most abundant genera in the FB + BS group were *UCG−002* (14.02%), *Christensenellaceae*_*unclassified* (9.98%), and *Ruminococcus* (9.09%), while the top three most abundant genera in the FB group were *Escherichia Shigella* (10.14%), *Muribaculaceae*_*unclassified* (9.55%), and *HT002* (9.36%) (Figure 4D).

## 4. Discussion

In this study, we found that adding *B. subtilis* to the feed of sows during late pregnancy and lactation can protect their offspring from *E. coli*-induced diarrhea by enhancing their immune response against *E. coli* and passing efficient maternal antibodies to their offspring. In addition, this treatment is expected to improve post-weaning growth in piglets by improving intestinal barrier function and flora composition.

*E. coli*-induced diarrhea in neonatal and weaned piglets results in significant economic losses for the pig industry. Due to the ongoing emergence of antibiotic resistance among *E. coli* isolates, alternative control measures, such as probiotics, organic acids, oral fimbria adhesins, feedback feeding, dietary zinc administration, and egg yolk antibodies, are increasingly recognized. However, despite these efforts, diarrhea caused by *E. coli* remains prevalent, and *E. coli* continues to be one of the primary pathogens associated with high morbidity and mortality in piglets [23,24].

In this study, a novel approach using probiotics (*B. subtilis*) combined with feedback feeding was established and implemented. We evaluated the effects of *B. subtilis* on the reproductive performance and maternal antibody levels in sows that were subjected to feedback feeding. Additionally, growth performance and diarrhea occurrence in their offspring were also monitored. As expected, there was no significant difference in the litter size between the two groups (*p* > 0.05). This is because *B. subtilis* was added to the feed on day 80 of gestation, rather than during the embryonic formation stage [25,26]. Moreover, *B. subtilis* did not affect the number of mummies (*p* > 0.05), possibly because these can be absorbed by the uterus during the middle-to-late stages of pregnancy [27]. In contrast to mummies, stillborn piglets are born dead, yet they resemble normal, living piglets [28]. In this study, we found that *B. subtilis* supplementation could decrease the number of stillborn piglets (*p* < 0.05), which is consistent with previous results [29,30,31]. Several studies have shown that probiotics can increase piglet weight at birth [29,32] by improving placental antioxidant capacity and increasing growth hormone concentrations in umbilical venous serum [32]. Usually, the number of live-born piglets and birth weight cannot increase simultaneously due to uterine space and resource constraints [33]. In this study, we found a significant difference in litter weight at birth (*p* < 0.05) but no significant difference in the average weight of live-born piglets (*p* > 0.05) between the two groups, which is consistent with previous studies [34,35]. 

Research has shown that the levels of specific IgA antibodies in milk are increased when the gut of sows is stimulated by antigens [36]. High levels of pathogen-specific maternal antibodies in sows stimulated by oral live or subunit vaccines are beneficial to the resistance of suckling piglets to external pathogens [37]. Studies have shown that feeding *B. subtilis* to sows can increase the levels of IgG, IgA, and IgM to a certain extent [38]. In this study, the levels of *E. coli*-specific maternal antibodies in the milk of sows in the FB group were found to be significantly increased in the FB + BS group (*p* < 0.05). A significant decrease in piglet diarrhea in the FB + BS group (*p* < 0.05) suggested that *B. subtilis* supplementation in sows treated with feedback feeding could protect their suckling piglets from *E. coli*-induced diarrhea by enhancing the levels of maternal antibodies. In addition, the pregnant mice (treated in the same way as sows) experiment further confirmed that IgA plasma cell accumulation in the intestines and IgA antibody transfer to the neonatal piglets via the gut–mammary axis might be the reasons why *B. subtilis* reduced neonatal porcine diarrhea caused by *E. coli*. Serum antibody level in piglets is highly correlated with that in sows, and SIgA is the main antibody that can be detected in intestinal contents [39,40]. In this study, we also found that the serum antibody level in piglets was positively correlated with that in sows due to the vertical transmission, which helps to improve the ability of piglets to resist diarrhea caused by pathogens such as *E. coli* or other stressors after weaning.

Besides the elevated maternal antibodies, an improved intestinal function may have contributed to the decreased diarrhea rate and weaning mortality in the suckling piglets born to the sows fed with *B. subtilis* and challenged with *E. coli*. The upregulation of tight junction proteins (*ZO*-*1*, *occludin*, *claudin1*) and *muc*-*2* indicated that *B. subtilis* improved intestinal barrier function in the piglets born to sows challenged with *E. coli*. Previous studies have indicated that colonization by intestinal microbes in suckling piglets is crucial to the maturation of intestinal function and the immune system [41,42]. Piglets are prone to significant changes in intestinal function due to changes in the nutritional psychological living environment during weaning, among which damage to intestinal flora composition is one of the key factors causing diarrhea in piglets [43]. Diversity in the flora composition enhances its ability to respond to gastrointestinal disturbances, and previous studies have clearly shown that the evenness index is a reliable indicator of microecological stability [44]. Our results demonstrated that combined feedback feeding and *B. subtilis* in sows induced a significant difference in the α and β diversity of the piglets’ intestinal flora. At the phylum level, the relative abundance of *Proteobacteria* in the FB + BS group was decreased, while, at the genus level, that of *UCG−002*, *Ruminococcus*, *Lactobacillus*, *Bacteroides*, and *Vibrio butyricum genera* in the FB group was increased. According to relevant studies, *Proteobacteria* include a variety of pathogenic bacteria, such as *E. coli*, *Salmonella*, *Vibrio cholerae*, etc., which are known as typical intestinal pathogenic bacteria. *Ruminococcus*, *Lactobacillus*, *Bacteroides*, and *Vibrio butyricum* are beneficial bacteria that are essential to polysaccharide metabolism after weaning. They can also generate short-chain fatty acids to inhibit the propagation of harmful bacteria [45,46] and activate intestinal immune function [47]. These results suggested that adding *B. subtilis* to the feed of sows may impact the abundance and composition of intestinal flora in their offspring through vertical transmission. 

Because the feedback feeding was a routine treatment on a farm, setting 2 groups of sows not receiving any intervention and a control group receiving *B. subtilis* alone without feedback was not allowed. This could be done in further studies using mice as the experimental model. As we all know, *B. subtilis* has the properties to alleviate oxidative stress and inflammation; whether it would through the improvement of pregnancy sows’ health improve their immune response remains to be further studied.

## 5. Conclusions

In this study, we found that adding *B. subtilis* to the feed of sows challenged with *E. coli* during late pregnancy and lactation could enhance the maternal immune response of sows to *E. coli*, which, in turn, led to better passive lactogenic immunity in their offspring, resulting in an increased number of weaned piglets, litter weight gain, immunity, intestinal barrier function, and intestinal flora composition, as well as a decreased *E. coli*-induced diarrhea rate.

## Figures and Tables

**Table 1:** Composition of the feed for pregnant and lactating sows.

Item	Lactating Sow Feed ^1^	Pregnant Sow Feed ^2^
composition g/kg		
Corn	705	650
Soybean pulp	175	160
Wheat bran	50.0	108.5
Soybean oil	8.5	25.0
Fish meal	20	15
Antioxidant	0.5	0.5
Mold inhibitor	1	1
4% Premix	40	40
nutritive value		
Metabolizable energy MJ/kg	14.02	30.01
Crude protein/%	18.0	12.8
Crude fiber/%	3.0	6.0
Lysine/%	1.1	0.4
Calcium/%	0.76	0.75
Phosphorus/%	0.62	0.60

^1^ Composition per kilogram of the lactating sow premix: vitamin A 120,000 IU, vitamin D31 15,000 IU, vitamin E 710 mg, vitamin K 32 mg, vitamin B12 5 mg, vitamin B26 25 mg, vitamin B63 5 mg, vitamin B3 35 mg, folate 15 mg, vitamin B5 4 mg, vitamin H 0.5 mg, Cu 35 mg, Mg 80 mg, Fe 80 mg, Mn 32 mg, Zn 101 mg, I 0.35 mg, Se 0.35 mg. ^2^ Composition per kilogram of the pregnant sow premix: vitamin A 220,000 IU, vitamin B2 105 mg, vitamin D3 65,000 IU, vitamin B6 70 mg, vitamin E 340 mg, vitamin K3 165 mg, vitamin B1 50 mg, vitamin B3 525 mg, vitamin B5 260 mg, I 10 mg, Fe 2600 mg, Zn 1000 mg, Mn 200 mg, Cu 175 mg, Se 11 mg.

**Table 2:** Primer sequences for qPCR assay.

Genes	Sequence of Forward and Reverse Primers
*ZO* *-* *1*	F: ATGAGCAGGTCCCGTCCCAAG R: GGCGGAGGCAGCGGTTTG
*occludin*	F: GACAGACTACACAACTGGCGG R: TGTACTCCTGCAGGCCACTG
*claudin 1*	F: CCATCGTCAGCACCGCACTG R: CGACACGCAGGACATCCACAG
*muc* *-* *2*	F: CTGTGCGACTACAACTTCGC R: AGATGGTGTCGTCCTTGACC
*β-actin*	F: CTGCGGCATCCACGAAACT R: AGGGCCGTGATCTCCTTCTG

**Table 3:** Effects of *B. subtilis* on the reproductive performance of sows challenged with *E. coli* and the growing performance of their offspring.

Items	FB ^2^	FB + BS ^2^	*p*-Value ^1^
sows			
Litter size (total births)	15 (2)	15 (4)	0.86
Litter weight at birth (kg)	16.78 ± 0.48	18.40 ± 0.44	0.02
Live-born piglet weight (kg)	1.29 ± 0.49	1.30 ± 0.52	0.83
piglets			
Number of weaned piglets	12 (1)	12 (2)	0.03
Litter weight at weaning (kg)	62.67 ± 0.75	67.40 ± 0.79	<0.001
Litter weight gain from birth to weaning (kg)	45.89 ± 0.90	49.00 ± 0.80	0.02
Piglet weight at weaning (kg)	5.45 ± 0.09	5.54 ± 0.07	0.44
Piglet daily weight gain from birth to weaning (g)	259.30 ± 4.48	263.76 ± 3.47	0.44
Piglet survival rate (%)	88.73 (173/195)	93.85 (183/195)	0.05
Diarrhea rate during lactation (%)	15.03 (26/173)	8.20 (15/183)	0.03

^1^ Values are means ± SD or medians (IQR). N = 15. ^2^ FB, feedback; FB + BS, feedback + *B. subtilis*.

## Data Availability

The data presented in this study are available from the corresponding author upon request.
